# Sex-Specific Differences in Kidney Function and Blood Pressure Regulation

**DOI:** 10.3390/ijms25168637

**Published:** 2024-08-08

**Authors:** Eleni Stamellou, Viktor Sterzer, Jessica Alam, Stefanos Roumeliotis, Vassilios Liakopoulos, Evangelia Dounousi

**Affiliations:** 1 Division of Nephrology and Clinical Immunology, RWTH Aachen University, 52074 Aachen, Germanyjalam@ukaachen.de (J.A.); 2 Department of Nephrology, University Hospital of Ioannina, 45500 Ioannina, Greece; edounous@uoi.gr; 3 2nd Department of Nephrology, AHEPA University Hospital Medical School, Aristotle University of Thessaloniki, 54124 Thessaloniki, Greece; roumeliotis@auth.gr (S.R.); vliak@auth.gr (V.L.)

**Keywords:** endothelin system, RAS, sex, androgen, estrogen

## Abstract

Premenopausal women generally exhibit lower blood pressure and a lower prevalence of hypertension than men of the same age, but these differences reverse postmenopause due to estrogen withdrawal. Sexual dimorphism has been described in different components of kidney physiology and pathophysiology, including the renin–angiotensin–aldosterone system, endothelin system, and tubular transporters. This review explores the sex-specific differences in kidney function and blood pressure regulation. Understanding these differences provides insights into potential therapeutic targets for managing hypertension and kidney diseases, considering the patient’s sex and hormonal status.

## 1. Introduction

Women live longer, experience a slower age-related decline in glomerular filtration rate (GFR), and are less prone to various kidney diseases [1]. One factor contributing to these differences may be the higher blood pressure observed in men. Starting at puberty, men develop higher blood pressure compared to women [2,3,4,5]. This trend reverses after menopause, when women’s blood pressure increases more rapidly and may eventually equal or surpass that of age-matched men [6]. Studies in both humans and animals indicate a higher prevalence and incidence of hypertension in males than females [5]. Consequently, higher blood pressure in men is associated with faster age-dependent decline in GFR, a more rapid progression of chronic kidney disease, and an increased risk of cardiovascular disease [7,8]. Interestingly, men with hypotestosteronemia or hyperestrogenemia exhibit a reduced incidence of coronary disease [9].

Animal models of hypertension, including *spontaneously hypertensive rats* (*SHRs*), *deoxycorticosterone acetate* (*DOCA*)-*salt*, and *chronic angiotensin* (*Ang II*) *infusion*, demonstrate that females are more resistant to blood pressure increases compared to males [10]. The correlation between these sex-specific blood pressure changes and hormonal alterations suggests that sex hormones might play a role in regulating blood pressure. Moreover, considering the kidney’s crucial role in blood pressure regulation, it is plausible that differences in blood pressure between the sexes are influenced not only by systemic sex hormones but also by kidney-specific hormonal actions. This review aims to explore the potential mechanisms underlying these phenomena. For this narrative review, PubMed was searched for publication using the term ‘gender differences’ or ‘androgens’, ‘sex hormones’, ‘sexual dimorphism’ and ‘kidney disease’ or ‘hypertension’, and hormonal influences. Of the articles that were found those published in languages other than English were deleted. Emphasis was placed on original works involving both humans (Table 1) and animals (Table 2).

## 2. Sex and Hypertension

Sexual dimorphism in hypertension has been observed in both human and laboratory animal studies, indicating that sex hormones significantly influence blood pressure regulation and related pathophysiological outcomes. For instance, studies on *Wistar rats* have shown that testosterone supplementation increases blood pressure in both male and female Wistar rats, an effect that can be blocked by the androgen receptor (AR) blocker flutamide, suggesting a direct role of AR in mediating hypertension [34]. Furthermore, in *SHRs* and in the *renal wrap model*, testosterone has been found to exacerbate hypertension, while flutamide effectively reduces blood pressure, highlighting the AR pathway’s involvement in salt-induced hypertension [21,22,23,35,36]. In *murine ren2 gene-expressing rats*, the administration of flutamide not only lowered blood pressure but also reduced organ damage, emphasizing the potential therapeutic benefits of AR blockade [24]. Prenatal exposure to testosterone cypionate in female rats resulted in elevated blood pressure and increased AR expression in the heart, suggesting that the early hormonal environment can have lasting effects on cardiovascular health [25]. Additionally, castration studies in various rodent models demonstrated a reduction in blood pressure, which could be reversed by testosterone supplementation, further underlining the role of testosterone in hypertension [21,27,37,38,39,40]. Interestingly, the impact of ovariectomy on blood pressure presents a contrast; while it does not affect blood pressure in female rodents, it leads to salt-independent hypertension in *Dahl salt-sensitive* (*DS*) *rats* [41,42]. This indicates a protective role of estrogens against hypertension, shedding light on the complex interplay between sex hormones and blood pressure regulation.

Sex hormones also influence the susceptibility to kidney damage. In a study, castrated rats on an 8% NaCl diet exhibited lower blood pressure, decreased albuminuria, plasma renin activity, and angiotensin concentrations, along with increased sodium excretion and creatinine clearance compared to controls. Testosterone supplementation reversed these effects [43]. Castration of young rats prevented the development of renal injury and hypertension. In 18-month-old male *SHRs* castrated at 8 months, blood pressure significantly reduced, glomerular injury was nearly absent, and GFR improved compared to their intact counterparts [30]. Male mice are more susceptible to ischemia/reperfusion (I/R) injury, with castration providing a protective effect [31]. Conversely, female sex hormones are believed to play a protective role in preventing renal injury [44,45,46,47]. Testosterone exacerbates glomerular injury in female *Sprague Dawley rats*, although the injury was still much milder than in the untreated male group [48].

While testosterone has some detrimental effects, it also has beneficial functions, particularly in males. Testosterone supplements in obese men improve insulin resistance, reduce body fat, and improve lipid profiles, without increasing blood pressure [49]. Low androgen levels in men are associated with various chronic diseases such as CKD, cancer, atherosclerosis, cardiovascular disease, sarcopenia, and osteoporosis [50,51,52]. However, it remains unclear whether the reduction in androgens is the cause or the result of these diseases [53]. Testosterone supplementation in male obese Zucker rats reduces metabolic syndrome symptoms but increases blood pressure [54]. In women, increased androgen levels lead to obesity and cardiovascular disease, and testosterone supplementation in a rat model of polycystic kidney disease increases cholesterol, leptin, and insulin levels while reducing glucose tolerance [13,55].

## 3. Kidney and Blood Pressure

Kidney dysfunction plays a critical role in the development and maintenance of hypertension, as shown by transplant studies in both humans and animals. Transplanting kidneys from prehypertensive individuals into normotensive recipients often leads to hypertension in the latter, indicating that blood pressure “follows” the kidney [56,57]. Similarly, kidneys from donors with a family history of hypertension significantly increase the recipient’s risk of hypertension compared to kidneys from donors without such a history [58]. Conversely, transplanting a kidney from a normotensive donor can normalize blood pressure in hypertensive patients [15]. Animal studies support this relationship. For instance, transplanting a kidney from *SHRs* to normotensive Wistar rats induces hypertension in the recipient. Transplanting a kidney from a normotensive to a hypertensive rat decreases the recipient’s blood pressure [59].

Sex hormones profoundly influence kidney function, affecting the organ’s structure, metabolic processes, and its ability to secrete and reabsorb substances. These hormones modulate the pharmacokinetic and toxicological responses to various compounds, largely due to hormonal regulation of specific kidney enzymes, ion channels, and membrane transport proteins [60,61,62,63]. Androgens are produced within the kidney, and ARs are found in different kidney tubule segments. Alterations in tubule transporter activity, driven by androgens, can result in sodium retention, which may lead to an increase in blood pressure [43,64,65,66,67,68]. This effect aligns with evidence showing that salt-induced hypertension is gender-dependent and the pressure–natriuresis response—the process by which the kidneys excrete sodium in response to blood pressure changes—is distinct between sexes. Specifically, females can excrete equivalent amounts of sodium at lower blood pressure compared to males, which is a difference observed in both humans and animal models [21,22,41,69,70,71]. Postmenopausal women become more susceptible to salt-sensitive hypertension and exhibit a more rapid progression of kidney diseases than premenopausal women, underscoring the critical role of the androgen–estrogen balance in blood pressure regulation [11,72,73]. Furthermore, androgens mediate hypertension through the sympathetic nervous system. Denervation prevents androgen-mediated blood pressure increase in hyperandrogenemic female rats (sympathetic nervous system) [74].

A transplantation study of Ang II-infused mice found that mean arterial pressure (MAP) increased significantly in female mice receiving a male kidney compared to female mice with a female kidney transplant. Male recipients of a female kidney had reduced MAP compared to the control group. Extrarenal regulation mechanisms also seem to influence the kidney, as angiotensin 1 receptor (AT1R) mRNA levels in the female-to-male transplantation group were increased by 50% compared to female-to-female mice, whereas AT1R levels in the male-to-female group were decreased compared to male-to-male mice. Additionally, endothelin-1 (ET-1) mRNA expression in female-to-male kidneys increased five-fold compared to the female-to-female group [75].

## 4. Endothelins

Endothelins (ETs), a group of endogenous peptides, mediate their actions through G protein-coupled receptors—endothelin A (ETA), endothelin B (ETB), and endothelin C (ETC). These peptides include endothelin 1 (ET-1), which is the more potent, endothelin 2 (ET-2), and endothelin 3 (ET-3). These receptors are distributed across various cell types within the cardiovascular system and kidneys, with ETA predominantly present on smooth muscle cells and ETB receptors located on endothelial and renal epithelial cells [76]. ET-2 and ET-2 are less studied and there are no data regarding a differential role in blood pressure regulation between sexes.

ET-1 exerts a wide range of biological effects in the kidney, including vasoconstriction of most renal vessels, mesangial cell contraction, enhancement of glomerular cell proliferation, induction of oxidative stress, expression of pro-inflammatory factors, and reduction of medullary blood flow through activation of ETA receptors. Conversely, activating renal ETB receptors induces vasodilation and inhibits Na+ and water reabsorption, resulting in subsequent natriuresis and diuresis [32,77,78]. The natriuretic effects of endothelin are primarily mediated via the ETB receptor by inhibiting Na+ reabsorption in the thick ascending limb and the collecting duct, or through vasodilation of vessels in the medulla during high salt intake, which enhances natriuresis and diuresis [32,79]. ETB receptors also influence vascular reactive oxygen species production and kidney nitric oxide synthase (NOS) activity, and they are involved in endothelin clearance, leading to its internalization and degradation. Collectively, ET-1 appears to have paradoxical effects on blood pressure regulation by promoting hypertension through vasoconstriction and relative hypotension through increased water and salt excretion [32,79,80]. Thus, the ET–ETB receptor pathway is generally seen as vasodilative and antihypertensive, in contrast to the prohypertensive ET–ETA pathway [81,82,83]. 

Sexual dimorphism in the ET-1 pathway is evident at various biological levels, including differences in plasma or tissue concentrations of ET-1, its production rates, receptor expression, physiological effects, and responses to receptor antagonists [58]. Evidence shows that plasma ET-1 levels are notably higher in men than in age-matched women. Moreover, in human aortic endothelial cells, ET-1 mRNA increases after stimulation with testosterone [84]. In contrast, estrogen appears to reduce ET-1 levels and prevent endothelin-mediated blood pressure elevation [85,86,87,88,89]. These differences seem to be hormonally regulated, as male-to-female transsexuals show lower ET levels after estrogen supplementation, while testosterone treatment in female-to-male transsexuals leads to an increase [12,78,90]. However, this hypothesis is challenged by findings that plasma ET-1 levels in males with hypogonadism are elevated, with a tendency to decrease after testosterone administration [91]. Furthermore, the contribution of ETB receptors to resting vascular tone differs between genders, with ETB receptors mediating tonic vasoconstriction in men, whereas in women, they mediate tonic vasodilation [92,93].

Deleting ET-1 or ETB receptor expression in collecting duct cells leads to salt retention and hypertension in mice [81]. The higher blood pressure elevation in male rats in the DOCA-salt model is presumably ETB receptor-mediated, as an ETB receptor knockout abolishes the blood pressure differences between the sexes [94]. Angiotensin II, whether stimulated exogenously or by a low-salt diet, inhibits ETB receptor function in male rats but to a much lesser degree in females [95]. Furthermore, acute infusion of an ETB agonist directly into the renal medulla enhances sodium and water excretion in both male and female rats. However, infusion of ET-1 only produces natriuresis in female rats, suggesting an altered functional distribution of ET receptors within the kidney [77]. This hypothesis is also supported by the different distribution of ET-1 receptors in the inner medullary collecting ducts of male and female rats, with females exhibiting fewer ETA receptors and an additional ETA-dependent natriuresis pathway potentially involving other cell types, such as the vasa recta, the thick ascending limb, or renomedullary interstitial cells. This excess of ETA receptors in males may inhibit ETB-mediated natriuresis [26]. This is supported by experiments in mice in which knocking out ET-1 and the ETB receptor led to salt retention and hypertension, whereas the knockout of the ETA receptor had no effect [96,97,98].

Female rats have alternative natriuretic mechanisms. Studies show effective salt excretion in ETB-deficient females, suggesting a prominent ET-independent salt excretion mechanism in females [99]. ETB-deficient female rats demonstrate higher medullary blood flow and enhanced diuresis and natriuresis following ET-1 infusion into the medulla, responses that are absent in male rats and diminished after ovariectomy [100,101]. The natriuretic response in male and ovariectomized female rats is diminished after ETA and ETB receptor blockade, highlighting a potential role for estrogens in this context [102]. Male ETB receptor-deficient rats on a normal salt diet also exhibit higher blood pressure and lower medullary NO activity than females. A high-salt diet further reduces NO plasma concentration and increases blood pressure, effects that are prevented by orchidectomy and reversed by testosterone supplementation [103]. Androgens in combination with a high-salt diet increase renal vascular resistance in male rats, whereas estradiol prevents salt-sensitive hypertension by enhancing NO production [104,105]. Conversely, female ETB receptor-deficient rats develop more severe hypertension on a high-salt diet than males, and gonadectomy increases the responsiveness of the afferent arteriole to ETB-induced vasoconstriction in females, but not males, indicating that female sex hormones may inhibit ETB-mediated vasoconstriction in the renal microcirculation [104,106].

ET-1 also contributes to kidney damage by increasing glomerular permeability for albumin and inducing glomerular oxidative stress, further exacerbated by high salt intake; these mechanisms are primarily ETA-mediated [107,108,109]. Nitric oxide (NO) production and pressure–natriuresis are decreased in mice with an ET-1 knockout in the collecting duct, suggesting that these processes are mediated by androgens, as ET-1 transgenic mice exhibit less renal injury post-castration [110,111]. Male DOCA-salt rats exhibit more severe kidney damage than females, but blocking the ETA receptor attenuates this damage in both sexes [90]. Studies shows higher renal ET-1 expression correlates with more severe kidney damage in male hypertensive rats. Estrogens seem to protect the kidney by lowering endothelin levels, as indicated by increased renal ET-1 expression and exacerbated renal injury after ovariectomy [112]. Estradiol suppresses kidney ET-1 production, which helps preventing acute kidney injury and other damage [113,114]. Male rats are more susceptible to renal ischemia/reperfusion injury, as male uninephrectomized rats had an 8% survival rate after 50 min of ischemia/reperfusion compared to 75% in females [31]. Orchidectomy reduces kidney damage and improves survival, with higher *ET-1* mRNA expression in males than females supporting this hypothesis [31,111].

Overall, these findings illustrate complex interactions between sex hormones, endothelin receptors, and their effects on renal physiology and blood pressure regulation, highlighting distinct differences in the pathophysiological responses between males and females. ET-1 expression, as well as renal damage markers, are higher in male than female hypertensive rats [90,115].

## 5. Tubule Transporters

Ion transporters across different segments of the kidney tubule exhibit sexual dimorphism, being partially modulated by androgens [66,67,116,117]. Sexual dimorphism in Na+ handling and androgen-dependent salt sensitivity have been reported [21,40,41,68,118,119]. Research by Veiras et al. demonstrated a sex-specific expression pattern of tubular ion transporters that results in more efficient sodium excretion in females [120]. Additionally, studies have noted an increase in Na+ excretion in the absence of testosterone [43]. The physiological consequences of increased Na+ reabsorption in the renal tubule are significant, not only in terms of blood pressure regulation but also in metabolic terms. Enhanced Na+ reabsorption increases oxygen consumption and promotes the production of free radicals within the kidney [28,121,122,123]. This increased oxidative stress can contribute to renal damage and exacerbate conditions such as hypertension, highlighting the complex interactions between ion transport, hormonal regulation, and kidney health.

### 5.1. Epithelial Sodium Channel (ENaC)

The epithelial sodium channel (ENaC) is one of the key salt transporters in the distal renal tubule. ENaC plays a pivotal role in sodium and fluid reabsorption in the renal tubule, influencing blood pressure regulation. Testosterone has been shown to increase the expression of the alpha subunit of ENaC (αENaC) through the AR in both rat models and the human renal cell line HKC-8 [29]. Treatment with testosterone propionate in orchidectomized Sprague Dawley rats upregulates the expression of all three units of *ENaC* in the kidney, an effect that is reversed by the administration of both flutamide and finasteride, suggesting the involvement of a 5-hydroxytestosterone-dependent pathway [124]. Other studies have reported an increase in renal *ENaC* expression in ovariectomized female *Wistar rats* and *SHRs* following dihydroxytestosterone administration [125]. In contrast, orchiectomy increases all three ENaC subunits’ expression in kidneys of male *Wistar rats* and *SHRs* [126].

Aldosterone, whose synthesis is also influenced by testosterone, further enhances *ENaC* expression in the kidney [127,128,129]. This relationship underscores a potential mechanism whereby testosterone increases blood pressure by promoting sodium retention through the upregulation of ENaC via the activation of the renin angiotensin system (RAS) pathway. Moreover, there are indications that ENaC regulation is also influenced by estradiol [130,131,132,133], suggesting a complex interplay between sex hormones and renal sodium handling that impacts blood pressure regulation. 

### 5.2. Sodium–Hydrogen Exchanger 3 (NHE3)

NHE3, located in the proximal tubule brush border membrane, plays a crucial role in sodium reabsorption and acid–base balance. Expression and activity of NHE3 was found to increase after an androgen-mediated upregulation of renal *Ang II* expression. This hormonal interaction leads to enhanced Na+ and fluid reabsorption, subsequently raising blood pressure [28,134]. The activity of the Na/H exchanger in the brush border membrane vesicles (BBMVs) is also stimulated by androgens, showing higher expression levels in males compared to females. Castration has been shown to decrease this activity, whereas testosterone supplementation reverses this effect, demonstrating the androgen dependency of this transporter system [135]. In male mice, castration leads to a decrease in Na/H+ exchanger expression, which is regulated by Ang II, further underscoring the interplay between androgens and renal hormonal pathways in regulating these transporters [135].

### 5.3. Aquaporins

Supplementation with testosterone of ovariectomized Wistar rats increased the expression of several aquaporins (*AQ*): *AQ1*, *AQ2*, *AQ4*, and *AQ7*, whereas *AQ3* was downregulated. The effects of testosterone were context-dependent; under normotensive conditions, *AQ3* expression decreased, but under hypertensive conditions, it increased. Expression of *AQ6* has also been shown to be increased by testosterone treatment under hypertensive conditions [136,137]. Further research supports these findings, showing that testosterone also increased blood pressure and similarly affected aquaporin expression in male Sprague Dawley rats, increasing levels of *AQ1*, *AQ2*, *AQ4*, *AQ6*, and *AQ7*, while simultaneously decreasing *AQ3* [138]. On the other hand, estradiol, a key estrogen hormone, appears to inhibit *AQ2* expression in the collecting duct, underscoring a potential contrasting role of estrogens in this regulatory pathway [139]. This interaction highlights the complex hormonal regulation of aquaporins, which plays a crucial role in the body’s fluid balance and blood pressure management. 

## 6. Renin–Angiotensin–Aldosterone System

Sex hormones play a significant role in regulating the renin–angiotensin system (RAS). Androgens increase the expression of several components of the RAS [53]. They stimulate the synthesis of angiotensinogen, with studies showing that castrated male rats have a 60% lower Ang II mRNA levels compared to controls [140,141]. Additionally, testosterone upregulates angiotensinogen levels in the kidneys of male SHRs and Sprague Dawley and DS rats [140,142,143]. Furthermore, there is a linear correlation between testosterone activity and plasma renin activity, with males showing higher plasma renin activity compared to females [144,145]. Testosterone treatment of ovariectomized rats results in increased plasma renin activity, while castration decreases it [140]. Acute testosterone treatment also increases plasma aldosterone levels [146].

Studies in SHRs have shown that testosterone promotes hypertension development via an androgen receptor-mediated mechanism that stimulates the systemic RAS. The final effector in the RAS, angiotensin II, promotes Na+ and water retention in the proximal tubule, contributing to higher blood pressure in males [147]. Ang II causes a greater increase in blood pressure in normotensive males than females (rats and mice) [148,149,150]. However, there is also a report that Ang II infusion had a similar effect on blood pressure in men and women on a controlled Na+ and protein diet [151,152]. The contribution of the RAS to gender-specific blood pressure differences could not be confirmed in aged heterozygous Ren-2 transgenic rats [42]. A possible explanation is the loss of the protective effect of estrogens in aged females as estrogen levels decline after menopause.

Besides the systemic RAS, the intrarenal RAS is a key component of blood pressure regulation, and sex steroids can modulate the expression of intrarenal RAS components [151,152,153,154]. The intrarenal RAS is more activated in males than in females and is downregulated after castration in DS rats and SHRs [128,143,155]. Androgens increase kidney angiotensinogen (AGT) levels significantly but have only a slight effect on liver AGT levels [140]. Angiotensinogen mRNA decreases in castrated male rats in the kidney and to a much lesser extent in the liver. Testosterone-treated castrates have the same angiotensinogen levels as the controls [140].

**Figure 1:**
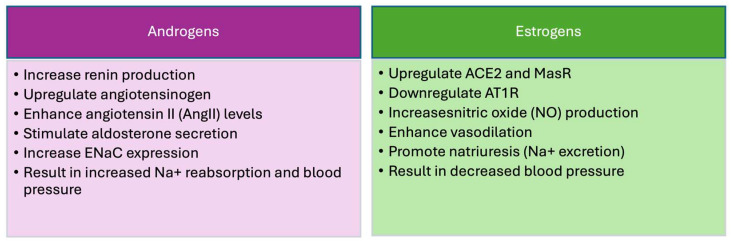
Sex-specific pathophysiological differences in kidney function and blood pressure regulation.

In contrast to androgens, estrogens induce the expression of non-classic RAS components that have a blood-pressure-lowering effect (Figure 1) [156,157]. This aligns with the observation that female normotensive rats have a higher *Ace2* and *Masr* gene expression than males [158,159]. Treatment with estrogen also decreases aldosterone production by upregulating the AT_2_R and downregulating the *AT1R* expression [160], thereby shifting the RAS towards blood-pressure-lowering pathways. Gupte et al. showed that estrogen protects female rats from hypertension by reducing the formation of Ang II in favor of Ang (1–7) in a high-fat diet (HFD) model [161]. Studies by another group revealed that estradiol downregulates the *AT1R* and ACE in the kidney, adrenal vasculature, and brain in female normotensive rats, thereby shifting the RAS towards non-canonical pathways [162]. It is also known that the *AT2R* is upregulated in female kidney of mice and rats, especially at high Ang II levels, and that females have a lower renal AT1R/AT2R ratio than males [33,163]. Ovariectomy, on the other hand, reduces the *AT2R* and upregulates the *AT1R* expression in SHRs [42]. This effect is reversed by estrogen administration. Castration of Sprague Dawley rats increases *AT2R* mRNA expression, which could be reversed by testosterone administration [164]. AT2R also reduces the sensitivity of the tubulo-glomerular feedback mechanism (regulator of GFR) only in females [165].

It also seems that there are some sex-specific differences regarding the response to treatment with the specific components of the RAS pathway. For example, treatment with angiotensin receptor blockers in women with congestive heart failure provides better survival rates than treatment with ACE inhibitors [16,166]. Interestingly, angiotensin receptor blockers reduce proteinuria in females more efficiently than in men [155]. Women also have a more rapid response to the AT1R antagonist irbesartan [151]. ACE inhibitors also reduce proteinuria in females more effectively than in males; however, blood pressure reduction by these agents seems to be similar in both genders [17,167,168].

Metabolites of arachidonic acid play a crucial role in kidney function and blood pressure regulation [169,170]. It has been shown that androgens can alter the metabolism of arachidonic acid in the renal cortex [169]. Androgen mediates production of 20-hydroxyeicosatetraenoic acid (20-HETE), an eicosanoid metabolite of arachidonic acid, which has varying effects depending on its site of expression. When *20-HETE* is expressed in the renal vasculature, it promotes vasoconstriction and hypertension. Conversely, if expressed in the kidney tubule, it prevents sodium reabsorption, leading to higher natriuresis and lower blood pressure [171]. Testosterone increases the expression of the *20-HETE* synthase Cyp4a12a in kidney of mice on a 129S6 background.

## 7. Sex-Specific Genetic Susceptibilities in Hypertension: Insights from GWAS

Even though several studies have reported both common and rare variants with a significant genome-wide association to hypertension [172,173,174,175,176], only a recent one identified genetic loci that have differential susceptibility to hypertension and blood pressure based on sex [18]. They demonstrate sex-specific genetic susceptibilities to hypertension. One notable finding is the *rs11066015* variant of the *ACAD10* gene, which showed exome-wide significance in males but not in sex-combined analyses. This variant, common in Asian populations, influences hypertension potentially through mitochondrial oxidative stress pathways. Furthermore, the *HECTD4* gene variants, *rs11066280* and *rs2074356*, displayed male-specific genetic significance, possibly linked to their role in the ubiquitin system and androgen receptor promotion. Similarly, the *rs67*1 variant of the *ALDH2* gene was associated with hypertension predominantly in men, likely due to its function in reducing oxidative stress. These sex-specific genetic effects suggest that hormonal differences may modulate these genetic pathways, contributing to the differential risk of hypertension between males and females.

## 8. Sex-Specific Nutraceutical Approaches

Evidence suggests that women and men may respond differently to dietary interventions due to sex hormones [177,178]. However, few controlled studies have assessed sex differences in cardiovascular response to specific diets [179]. For example, adherence to a Mediterranean diet (MedDiet) has been shown to significantly lower cardiovascular disease (CVD) risk more in women than in men [179]. Regarding hypertension, epidemiological data indicate that whole grains and legumes reduce hypertension risk in women, but not in men, even after controlling for confounders [19]. In the Korean National Health and Nutrition Examination Survey, women who frequently consumed fried foods (≥2 times/week) had a 2.4-fold higher risk of hypertension compared to those who rarely consumed fried foods, a significant association not seen in men [20]. Additionally, reductions in blood pressure with diets low in sodium, and those supplemented with potassium or dark chocolate, were greater in women than in men [14]. A healthy diet was more effective in alleviating hypertension risk factors in women than in men [180].

Overall, the existing literature underscores the importance of investigating sex-related differences in dietary responses. Such research could lead to individualized dietary recommendations, improving health outcomes for both men and women. 

## 9. Conclusions

**Figure 2:**
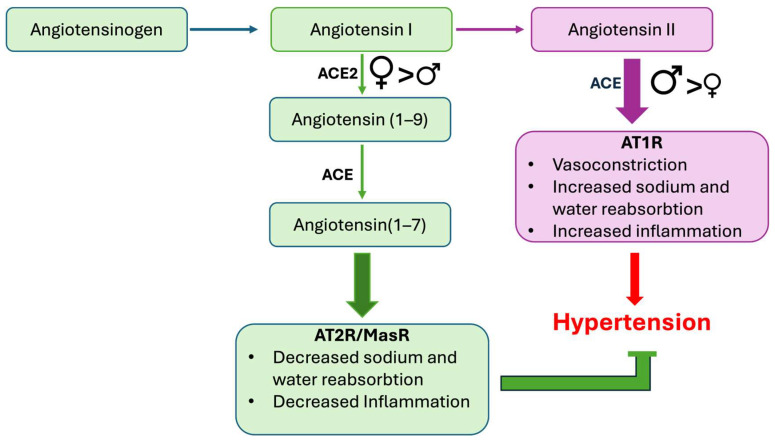
Overview of suggested sex differences in the renin–angiotensin–aldosterone system. Males typically have a more active ACE/Angiotensin II/AT1R pathway, increasing the risk of hypertension, while females tend to have a more active AT2R/MasR pathway, offering protective effects against hypertension.

This review highlights the significant impact of androgens on kidney function and blood pressure regulation, emphasizing the sex-specific differences mediated by these hormones (Figure 2). Understanding these mechanisms provides insights into potential therapeutic targets for managing hypertension and kidney diseases, considering the patient’s sex and hormonal status. Future research should focus on elucidating the precise molecular interactions and long-term impacts of sex hormones on renal and cardiovascular health, paving the way for more personalized treatment strategies.

## Figures and Tables

**Table 1:** Important human studies on sexual dimorphism in kidney disease and hypertension.

Study	Population	Key Findings
Messerli, F.H. et al. [11]	Male and female patients with hypertension	Found that men have higher blood pressure compared to women starting at puberty, with this trend reversing after menopause.
van Kesteren, P.J.M [12]	M—>F and F→M transexuals	Male-to-female transsexuals exhibit lower ET levels after estrogen supplementation, whereas testosterone treatment in female-to-male transsexuals leads to an increase in ET levels.
Phillips, G.B. et al. [9]	Men with coronary artery disease	Found an association of hypotestosteronemia with coronary artery disease in men.
Christakou, C.D. et al. [13]	Women with polycystic ovary syndrome	Discussed the role of androgen excess on metabolic aberrations and cardiovascular risk in women with PCOS.
Chen, W. et al. [14]	Men and women without hypertension at baseline	Suggested that the endothelial NO synthase gene may contribute to the predisposition of females for hypertension
Curtis, J.J. et al. [15]	Hypertensive patients undergoing renal transplantation	Found remission of essential hypertension after renal transplantation.
Zapater P. et al. [16]	Men and women with hypertension	Found that women had lower blood pressure and angiotensin converting enzyme (ACE) activities than men
Ruggenenti P. et al. [17]	Men and women with hypertension	Found that men exhibit a lower response to ACE inhibitor treatment
		
Cho, S.B. et al. [18]	GWAS	Indicated a sex-specific genetic susceptibility to hypertension
Song, S. et al. [19]	Men and women without hypertension at baseline	Found that a diet rich in grains and legumes is inversely associated with the risk of hypertension in Korean women
Kang, Y. et al. [20]	Men and women without hypertension at baseline	Found that frequent fried food consumption is associated with hypertension in Korean women

**Table 2:** Sex-specific findings in various animal models of hypertension.

Animal Model	Sex-Specific Findings	References
Spontaneously Hypertensive Rats (SHRs)	Testosterone mediates hypertension via AR; flutamide decreases blood pressure	[21,22,23,24,25]
Deoxycorticosterone Acetate (DOCA)-Salt Rats	Higher blood pressure elevation in males; *Endothelin B* (*ETB*) receptor knockout abolishes blood pressure differences	[26]
*Ren2* Gene-Expressing Rats	Flutamide lowers blood pressure and reduces organ damage	[27]
Orchidectomized Sprague Dawley Rats	Testosterone increases *ENaC* expression; castration decreases blood pressure	[28,29]
Ovariectomized Rats	Salt-independent hypertension in *DS rats* post-ovariectomy	[30,31]
Angiotensin II (Ang II)-Infused Mice	Blood pressure increase in females with male kidney; *Angiotensing 1 receptor* (*AT1R*) and *endothelin 1* (*ET-1*) mRNA differences	[32]
High-Fat Diet (HFD)-Induced Hypertension	HFD induces hypertension in males; losartan prevents this; and females resistant due to Ang (1–7)	[33]
